# Cross-talk of Three Molecular Subtypes of Telomere Maintenance Defines Clinical Characteristics and Tumor Microenvironment in Gastric Cancer

**DOI:** 10.7150/jca.92207

**Published:** 2024-04-15

**Authors:** Mengpei Yan, Zhijun Zhang, Luyao Wang, Hongxin Huang, Jihuan Wang, Chengjun Zhu, Zheng Li, Zekuan Xu

**Affiliations:** 1 Department of General Surgery, The First Affiliated Hospital of Nanjing Medical University, Nanjing, 210029, Jiangsu Province, China.; 2 Collaborative Innovation Center for Cancer Personalized Medicine, Nanjing Medical University, Nanjing, Jiangsu Province, China.; 3 The Institute of Gastric Cancer, Nanjing Medical University, Nanjing, Jiangsu Province, China.

**Keywords:** telomere maintenance, gastric cancer, tumor immune infiltration, molecular subtypes, TUBB6

## Abstract

**Background:** Telomere maintenance takes part in the regulation of gastric cancer (GC) pathogenesis and is essential for patients' clinical features. Though the correlation between a single telomere maintenance-related gene and GC has previously been published, comprehensive exploration and systematic analysis remain to be studied. Our study is aimed at determining telomere maintenance-related molecular subtypes and examining their role in GC.

**Methods:** By analyzing the transcriptome data, we identified three telomere maintenance-associated clusters (TMCs) with heterogeneity in clinical features and tumor microenvironment (TME). Then, we screened five prognostic telomere maintenance-related genes and established corresponding TM scores. Additionally, the expression level and biological function of tubulin beta 6 class V (TUBB6) were validated in GC tissues and cells.

**Results:** TMC1 was correlated with EMT and TGF-beta pathway and predicted low tumor mutation burden (TMB) as well as bad prognostic outcomes. TMC3 was associated with cell cycle and DNA repair. In terms of TMB and overall survival, TMC3 exhibited opposite results against TMC1. Significant heterogeneity was observed between TMCs. TUBB6 was upregulated and could promote GC proliferation, migration, and invasion.

**Conclusion:** Altogether, combining bioinformatics and functional experiments, we identified three molecular subtypes based on telomere maintenance-associated genes in GC, which could bring new ideas and novel biomarkers to the clinic.

## Introduction

As one of the most prevalent malignant tumors, gastric cancer (GC) resulted in the third most cancer-related deaths all over the world 1. Despite increasingly refined tumor-node-metastasis (TNM) staging systems of GC subtypes and rapid developments in GC treatment 2, there remained large numbers of GC patients that have obtained limited benefits from current mainstream cancer treatments, such as surgical resection, chemotherapy, targeted therapies, and so on 3. Imminent importance was attached to seeking new biomarkers and setting up innovative classification systems.

As is known to all, there have been significant advancements in the exploration of therapeutics in order to interfere with the replication progress of cancer cells 4, 5. As the key structure related to replicative potential, telomeres consisted of repeated tandem arrays of (TTAGGG)_n_ at the distal ends of chromosomes and restricted the lifespan of cells by shortening with every cell division 6, 7. Telomere erosion was known to construct the complex mechanism regulating the cell aging process together with normal development, mutations, and epi-mutations 8. Telomere shortening could be compensated by telomerase and alternative lengthening of telomeres (ALT) 9, 10. The vast majority of cancer cells overexpress telomerase 11, 12, while abnormal activation of ALT has been observed in some telomerase-negative tumors activating ALT 10, 13. A multitude of studies have provided theoretical evidence for further exploration of telomere maintenance.

Telomere maintenance (TM) has been reported to play roles in GC in a complex manner. The expression of telomeric repeat binding factors and TRF1-interacting nuclear protein 2 in GC has been validated in the early years 14, 15. In addition, inhibition of tankyrase 1 could shorten telomere length and co-inhibition of tankyrase 1 and telomerase may be a potential therapy for telomere-directed GC 16, 17. Recent single-cell analysis of GC reveals that non-defined telomere maintenance mechanism type cells maintained their survival, proliferation, and homeostasis by regulating the microenvironments 18. Meanwhile, the immune microenvironment of tumor cells has been published to play regulatory roles in GC patients' prognosis and has attracted the attention of many researchers 19. However, there is still a lack of the establishment of an evaluation model or molecular subtypes for telomere maintenance-related gene signatures on the clinical features of GC and corresponding studies about the association of telomere maintenance with the immune microenvironment, which reveals the necessity of our study.

## Methods

### Datasets

Our study contained a total of 1310 STAD patients from various databases. Four public datasets, including TCGA STAD (385 GC patients and 35 normal participants), GSE15459 (192 GC patients), GSE66229 (300 GC patients), and GSE84437 (433 GC patients), were involved in our research, while the patients without survival status or survival time were excluded from our research. The common clinical features enrolled in the study included “Dataset”, “OS”, “OS.time”, “Age”, “Gender”, “Stage”, “T_stage”, “N_stage”, “M_stage”, “TP53_mutation”, and “KRAS_mutation”, which could be available in the supplementary material (Table S1, S2). The RNA-seq transcriptome data of GC patients in the TCGA database was obtained in the manner of transcripts per kilobase million (TPM). We combined three GEO datasets (GSE15459, GSE66229, GSE84437) and built a meta-GEO dataset. We used “Combat” R package to eliminate the batch effect. Our simultaneous search of the TelNet database (https://malone2.bioquant.uni-heidelberg.de/fmi/webd/TelNet) yielded 2093 genes relevant to telomere maintenance. Corresponding somatic mutation data were downloaded from the TCGA STAD cohort.

### WGCNA

To further eliminate the genes most connected to TM, we used the “WGCNA” R package to carry out this part. We decided on 3 as the soft power and acquired 8 modules for correlation analysis with TM and additional clinical traits. The turquoise and yellow modules were screened out as the final gene modules for further analysis (Table S3).

### Consensus clustering analysis

The two selected gene modules were delivered to univariable Cox regression for further clustering (Table S4). The “ConsensusClusterPlus” R package was used to categorize STAD patients on the basis of the expression patterns of telomere maintenance-related genes (TMGs). We determined the proper number of the clustering by cumulative distribution function (CDF) curves, delta area, and the heatmap of the cluster. Kaplan-Meier curves were plotted to display the overall survival of each telomere maintenance-associated cluster (TMC) with the “survival” and the “survminer” R packages. We performed the same procedure with differentially expressed genes (DEGs) between the TMCs in order to identify gene clusters. The “limma” R package was applied to screen DEGs between different clusters.

### Gene set variation analysis (GSVA) and single-sample gene set enrichment analysis (ssGSEA)

By the use of the “GSVA” R package, we evaluated the differences in pathways enriched by each cluster in the manner of the GSVA method. Corresponding gene sets were gained from the MSigDB Team v2023.1 and the “limma” R package was applied to find significant pathways. The comparability of multiple pathways including TM was realized in each sample with the ssGSEA. Built with previous studies 20, markers of different cell types were delivered to the ssGSEA scoring system, and the “CMScaller” R package was employed.

### Evaluation of TME among different TMCs

Three algorithms (ssGSEA, Cibersort, and Estimate) were used to evaluate TME among different TMCs. We calculated the ssGSEA score of various cell types according to previous research. The proportion of each immune cell was analyzed with Cibersort analysis. The estimate algorithm consisted of the stromal score, immune score, and combined score.

### Establishment of the TM score and prognostic analysis

Followed by LASSO regression to reduce dimensionality, Multivariable Cox regression was operated to screen prognosis-related genes. Five genes were identified to construct the model. All patients were divided into low TM score (TMs_score < median value) and high TM score (TMs_score > median value) subgroups. We performed KM analysis to analyze the differences in OS between the two subgroups and the “survivalROC” R package was used to create the time-dependent receiver operating characteristic (ROC) curve. The model was built as:

TMs_score = ∑^i^_n = 1_ (Coefi * GeneExp)

### Tumor mutation burden, drug susceptibility, and clinical correlation analysis

The somatic mutations of GC patients were divided into two groups in accordance with different TM scores by the use of the “maftools” R package. The”pRRophetic” program was used to examine the semi-inhibitory concentration (IC50) values of chemotherapeutic drugs routinely applied to treat GC patients. The correlations between the TMs_score and the clinical characteristics were compared with chi-square testing.

### Cell culture and siRNA transfection

The cell lines involved in our study were purchased from the Cell Center of Shanghai Institutes for Biological Sciences. We cultured AGS cells in Nutrient Mixture F-12K medium (Wisent, Canada), while the other cells were cultured in RPMI 1640 medium (Wisent, Canada) in a humidified atmosphere of 5% CO2 at 37 °C. All medium was added with fetal bovine serum (FBS; 10%, Wisent, Canada) together with 1% penicillin-streptomycin.

Small interfering RNAs (siRNAs) designed against tubulin beta 6 class V (TUBB6) (GenePharma, China) and their negative control were synthesized according to previous studies 21, 22. Lipofectamine 3000 (Invitrogen, USA) was made use of for transfection. The sequences of siRNAs were as follows: si-1: 5'-GAGAGAAUCAACGUCUACU-3'; si-2: 5'-CGAAAGGGCACUACACGGA-3'.

### Tissue samples

After obtaining informed consent, we selected eighty GC tissues and their adjacent normal tissues from the First Affiliated Hospital of Nanjing Medical University between April 2016 and April 2022. All subjects have obtained definitive diagnoses by professional pathologists and have not undergone preoperative chemoradiotherapy or other malignancies. We collected relevant clinical information from medical records. The Ethics Committee of the First Affiliated Hospital of Nanjing Medical University has approved our study.

### RNA extraction and quantitative real-time PCR (qRT-PCR)

Trizol Reagent (Invitrogen, Carlsbad, CA, USA) was employed to extract RNA from the aforementioned cells and tissues. After reverse transcription, we performed qRT-PCR (ABI 7300) to evaluate the expression of corresponding mRNA, and the results were presented by the 2-ΔΔCT method. The primers are as listed: TUBB6 forward, 5'-GCAAATTAGGAGGGAGTTAG-3' and TUBB6 reverse, 5'-GCATATTCATATAAGGCAACAC-3'; GAPDH forward, 5'-TGCACCACCAACTGCTTAGC-3' and GAPDH reverse, 5'-GGCATGGACTGTGGTCATGAG-3'.

### Functional experiments

We used MKN45 and AGS cells to perform functional experiments including CCK-8 assay, clonogenic assay, wound-healing assay, and transwell assay *in vitro* following the protocols introduced previously by us 23.

### Statistical analysis

The Wilcoxon rank-sum test was conducted to evaluate differences between the two groups, while the Kruskal-Wallis H test was used to compare groups of three or more. Univariate and multivariate Cox regression were used to seek independent prognostic factors. Association and survival analyses were conducted by Pearson and log-rank test. Each experiment was performed in both technical and biological triplicate for precision and rigor. All statistical analyses were performed in R 4.2.1 and two-tailed p < 0.05 was defined as statistically significant.

## Results

### Identification of telomere maintenance-related subtypes

**Figure 1:**
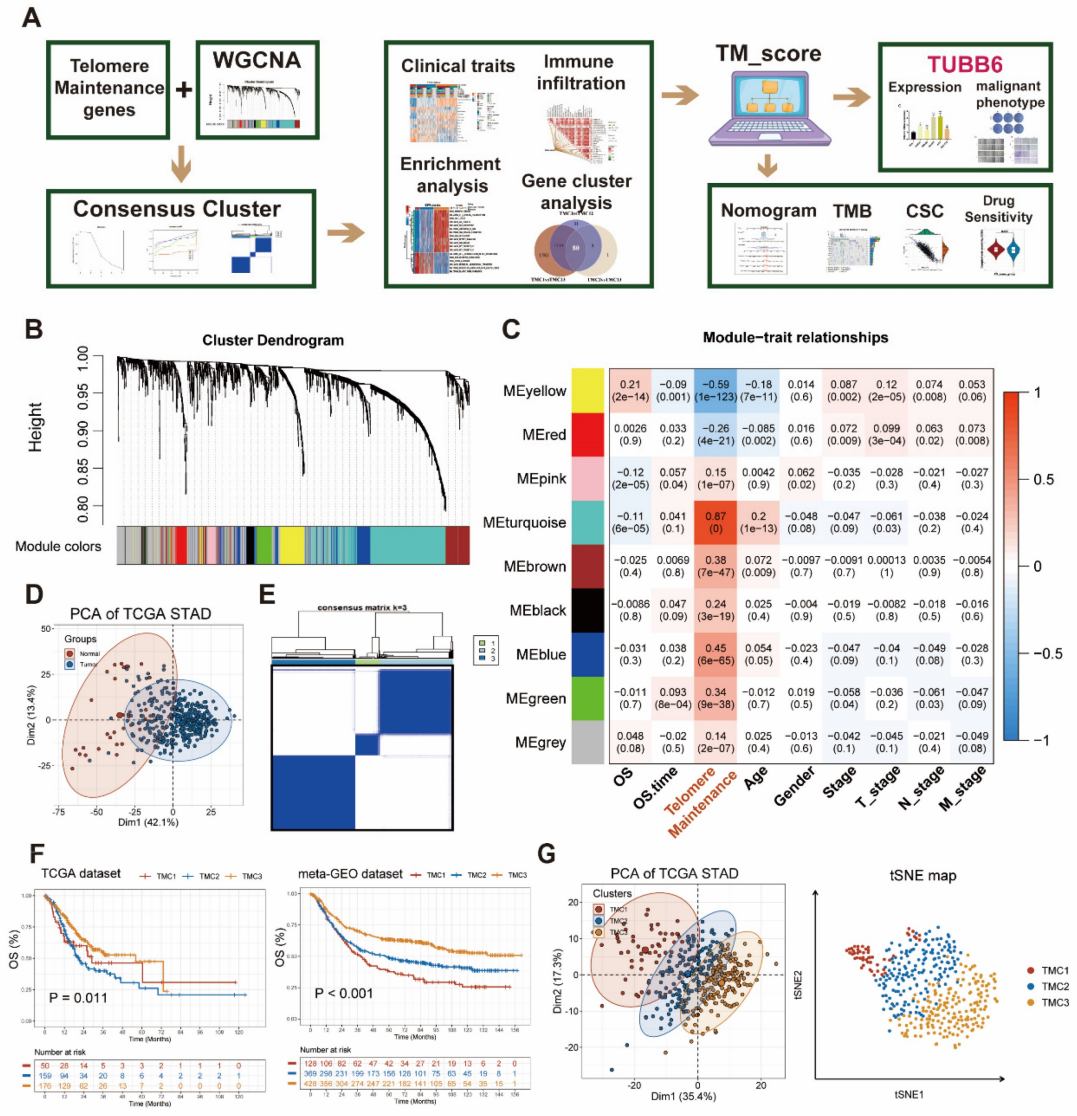
** Identification of genes most related to telomere maintenance (TM) and construction of TM related clusters (TMCs). (A)** The flow chart of the whole article. **(B)** The 282 TM-related genes screened by univariate Cox regression were clustered into multiple modules with WGCNA. **(C)** Correlation analysis between gene modules and traits to screen out the most relevant modules with TM. **(D)** TM-related genes were effective at differentiating between malignant and normal tissues. **(E)** Consensus cluster analysis identified three clusters using 282 TM genes. **(F)** Survival analysis highlighted TM cluster-specific prognostic differences. **(G)** TM clusters could be recognized, according to PCA and tSNE plots.

The whole flowchart diagram of our study was shown (Figure 1A). The clinical traits of all patients were shown in Table S1. Telomere maintenance-related genes (TMGs) were obtained from the TelNet database and the gene modules related to TM were acquired using the WGCNA. Following clustering genes with similar expression patterns into co-expression modules (Figure 1B), we analyzed the relationships between each gene module and clinical and biological traits (Figure 1C, Figure S1A-B). The two modules (turquoise and yellow) (Table S3) associated most significantly with telomere maintenance were chosen to conduct subsequent studies. Univariable Cox analysis was performed to achieve the final 282 TMGs (Table S4). The PCA map was used to visualize the differences in TMGs between normal and tumor tissues from the TCGA STAD database (Figure 1D), which indicated that it was quite effective to separate tumors from normal tissue with TMGs. For a deeper understanding of these genes, we categorized GC patients into three telomere maintenance-associated clusters (TMCs) by the use of the consensus clustering analysis (Figure 1E, Figure S1C-G, Table S1). Figure S1H provided a corresponding standard for the construction of clusters as well. The comparisons of clinical factors of the three TMCs were displayed in Table S2. In order to evaluate the effects different TMCs have on prognosis, we plotted Kaplan-Meier survival curves according to the TCGA database and the meta-GEO dataset, respectively. In both the TCGA database and the meta-GEO dataset, TMC1 had the worst prognosis and TMC3 had the contrary results (Figure 1F). Additionally, TMC heterogeneity was explored separately with a PCA map and t-SNE analysis and we achieved significant results in both two ways (Figure 1G).

### Discrepancies among TMCs in clinical features and biological pathways

**Figure 2:**
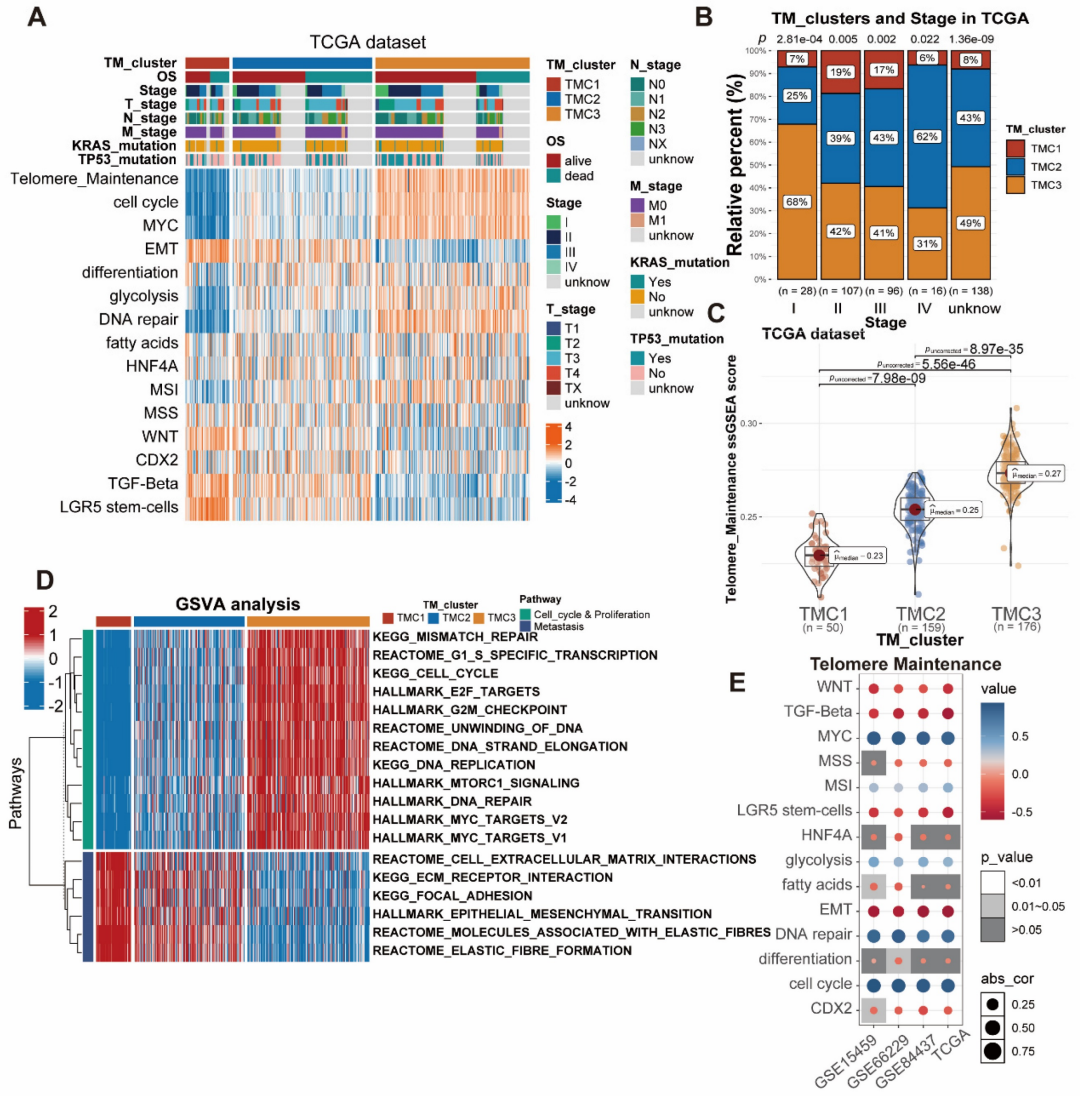
** Investigation of the three TMCs in biological and clinical traits. (A)** The distinctions between TMCs were discovered by examining clinical characteristics and 15 pathways assessed by ssGSEA. **(B)** Proportion of TMCs in different clinical stages. **(C)** TMCs' respective TM ssGSEA scores uncovered that TMC3 scored the highest while TMC1 the lowest. **(D)** GSVA analysis displayed the difference between TMCs in metastasis and proliferation. **(E)** Bubble plots demonstrated the relationship between the TM and additional biological pathways in various data sets.

The associations between distinct TMCs and clinical features were further explored by us. In brief, TMC1 was associated with the worst prognosis, the worst telomere maintenance, and metastasis pathways, while TMC3 predicted the opposite effects in the prognosis and the telomere maintenance. In addition, TMC3 was related to the cell cycle, DNA repair, and metabolism pathways. The characteristics of TMC2 were between TMC1 and TMC3. The heatmap presented that a strong positive correlation was observed between EMT, TGF-beta, and TMC1, while the opposite relationship was shown between telomere maintenance, cell cycle, MYC, DNA repair, and TMC1. Meanwhile, TMC3 was positively correlated with telomere maintenance, cell cycle, MYC as well as DNA repair, and negatively correlated with EMT (Figure 2A, Figure S3A-B). GO analysis supported the above results (Figure S2A-C). Furthermore, 385 patients from the TCGA STAD database were divided into five groups anchored in their pathologic stage. The analysis showed that the proportion of TMC3 decreased with the advanced stage, and the opposite trend was observed in that of TMC2 (Figure 2B). Similar results were observed in the meta-GEO database as well (Figure S3C). As the telomere maintenance score was quantified with ssGSEA analysis, we discovered that TMC1 had a relatively lower telomere maintenance score than TMC3, which implied that the lower level of telomere maintenance score may predict a worse prognosis (Figure 1F, Figure 2C, Figure S3D). Thence, we tried to explore the influence of telomere maintenance on biological pathways. GSVA analysis revealed that TMC1 was bound up with metastasis, whereas TMC3 was related to cell cycle and proliferation (Figure 2D, Figure S3B). The correlation analysis based on different datasets also demonstrated this consequence (Figure 2E).

### Associations between tumor microenvironment and TMCs

**Figure 3:**
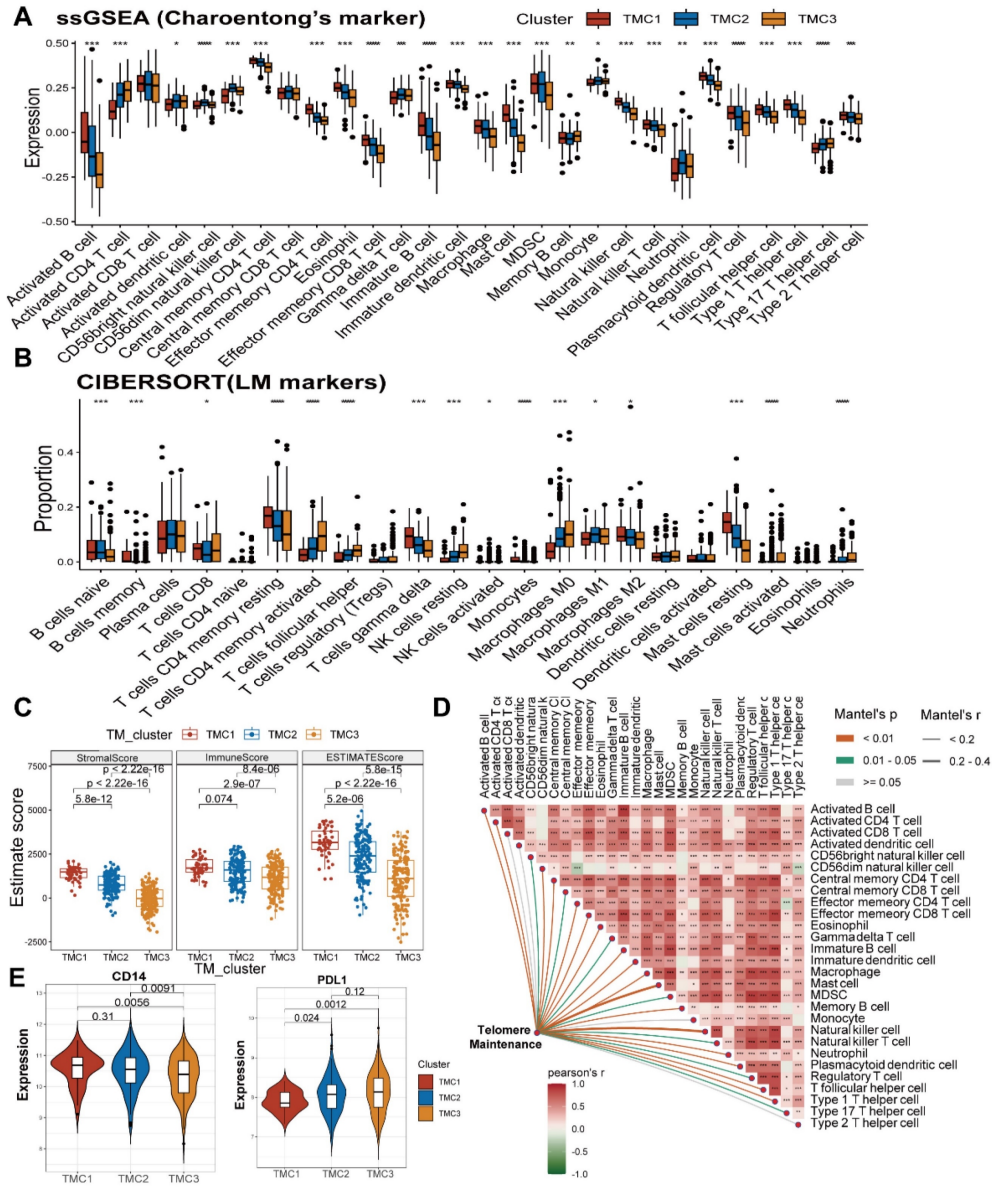
** Exploration of TME in TMCs. (A)** ssGSEA analysis revealed the infiltration of different immune cells in TMCs using Charoentong's cell markers. **(B)** Cibersort analysis uncovered the Immune cells' proportion in TMCs using LM cell markers. **(C)** ESTIMATE algorithm assessed immune and stromal heterogeneity in TMCs. **(D)** TM was calculated using association analysis on different immune cells. **(E)** Differential expression of PDL1 and CD14 in TMCs (* p <0.05, ** p <0.01, *** p <0.001).

Two groups of TME cell signatures were accessed to perform ssGSEA and cibersort analyses. The first set of outcomes suggested that more activated CD4 T cells and fewer natural killing cells were observed in TMC3 and TMC2 than that in TMC1. Additionally, the aggregation of regulatory T cells among TMCs exhibited extremely significant differences (Figure 3A, Figure S4A). In the other signature, different types of macrophages (M0, M1, and M2) had different distributions among TMCs (Figure 3B, Figure S4B). Moreover, built with the Estimate methodology, TMC1 had remarkably higher estimate scores relative to TMC3, which was attributed mostly to the high stromal scores of TMC1 (Figure 3C, Figure S4C). We found that telomere maintenance was interrelated to natural killer cells after analyzing the correlations between telomere maintenance and each TME cell (Figure 3D, Figure S4D). Then, the two most significant immune markers, CD14 and PDL1 were chosen to conduct further analysis. We observed higher expression of CD14 in TMC1, while TMC3 expressed relatively lower PDL1 (Figure 3E, Figure S4E). Above all, these consequences highlighted the heterogeneity of immune states in various TMCs.

### Identification and exploration of TMG clusters

**Figure 4:**
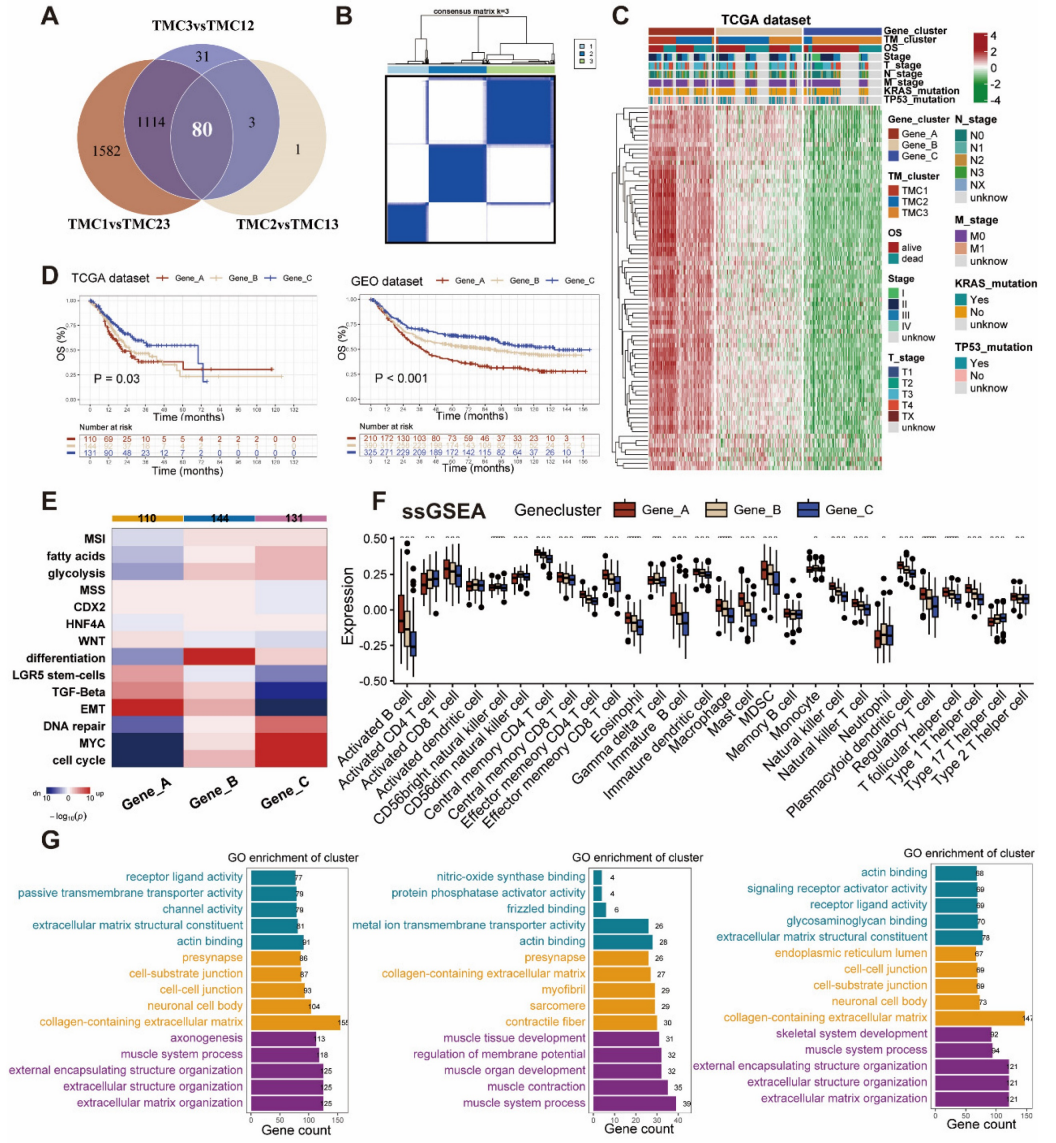
** Construction and exploration of gene clusters related to TMCs. (A)** Venn plot showed the intersection genes of DEGs of TMCs. **(B)** Consensus cluster analysis recognized three gene clusters with the 80 DEGs. **(C)** Heatmap of the three gene clusters revealed variations in clinical and DEG expression. **(D)** Survival plots revealed diffenrences in three gene clusters in both TCGA and GEO datasets. **(E)** Functional enrichment analyses were performed in the three gene clusters. **(F)** ssGSEA analysis revealed the infiltration of different immune cells in gene clusters. **(G)** GO analysis were conducted in the three gene clusters, respectively. (* p <0.05, ** p <0.01, *** p <0.001).

Eighty differentially expressed genes were identified by taking the intersection of that between each pair of TMCs (Figure 4A, Table S6). The aforementioned genes were classified into three clusters by the use of consensus clustering (Figure 4B). We analyzed the expression pattern and clinicopathologic features of distinct gene clusters were exhibited in the manner of a heatmap (Figure 4C). To figure out the prognostic effects of different gene clusters, we plotted Kaplan-Meier survival curves and found that gene cluster A predicts the worst prognosis and gene cluster C foreshadowed the best overall survival in both TCGA and meta-GEO datasets (Figure 4D). Enrichment analysis revealed that the gene cluster A promoted TGF-beta as well as EMT, and inhibited DNA repair, cell cycle, and MYC. Gene cluster C seemed to play an opposite role in the biological pathways mentioned above (Figure 4E). The genes in cluster A were expressed highly in the majority of TME cells in accordance with the ssGSEA signatures (Figure 4F). Subsequently, we conducted GO analysis to explore the pathways related to different gene clusters, uncovering Gene cluster A was mostly enriched in metastasis (Figure 4G).

### Effects of TMGs on GC prognosis

**Figure 5:**
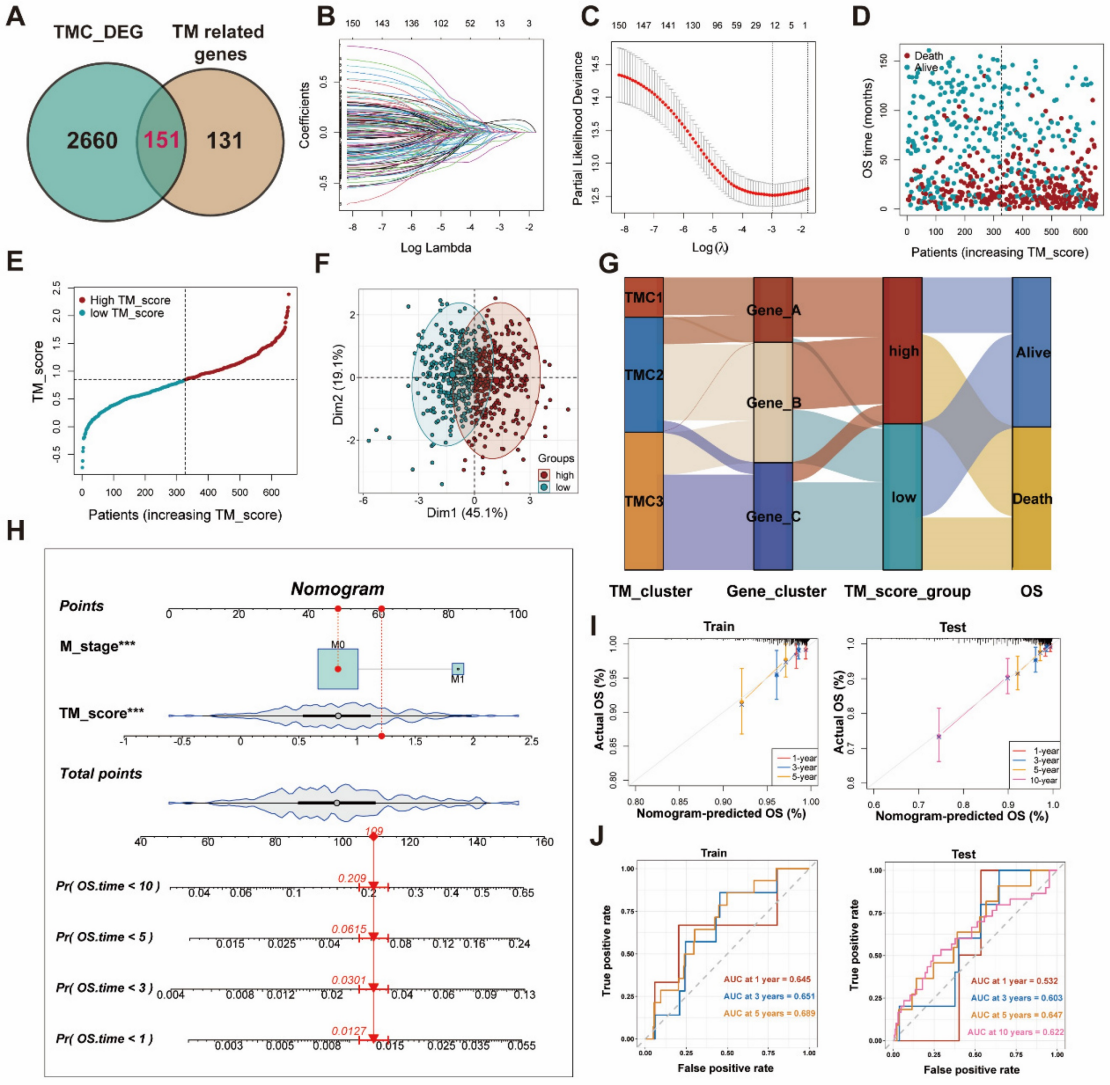
** Establishment of TM score in train set for clinical application. (A)** Screening genes to construct lasso regression and multivariate cox models. **(B-C)** Lasso regression analysis identify 12 genes for multivariate Cox regression. **(D-E)** Distribution of TM scores in patients with different OS status. **(F)** PCA analysis exhibited the distribution between the high and low TM score groups. **(G)** The sankey plot displayed the flow of different clusters for each patient. **(H)** Construction of the nomogram. **(I)** The calibration curves for the nomogram in train and test sets. **(J)** The AUC curves for the nomogram in train and test sets.

All patients were randomly divided into train (654 patients) and test (656 patients) sets for modeling and validation. The clinic parameters of patients in the train and test cohorts have been analyzed and no statistical differences were found between them, which was mentioned in Table S5. After filtering out prognosis-related genes from TM-related genes (Table S4), we took the intersection of these genes and differentially expressed genes among TMCs (Figure 5A, Table S6). Five genes were finally identified with LASSO regression analysis followed by multiple logistic regression analyses (Figure 5B-C, Table S7). Then, the correlation evaluation between the TM scores of GC patients and their overall survival showed that the higher TM score may predict a worse prognosis in GC patients of both train and test sets (Figure 5D, Figure S5A). Afterward, we found that the distribution of TM scores among patients was uniform and corresponding patients could be divided into two groups according to the TM score (Figure 5E-F, Figure S5B-C). The relationship between clusters, TM scores, and survival state was visualized by the alluvial plot. A high proportion of gene cluster A was observed in TMC1, the majority of whom had high TM scores and bad prognosis, while most of the patients in TMC3 expressed a high level of gene cluster C, got low TM scores, and seemed to have better outcomes (Figure 5G). The 1-, 3-, 5-, and 10-year OS time was evaluated for the patients on the basis of the nomogram established on the M stage and TM score (Figure 5H), which was the optimal combination. In addition, aimed at validating the precision of the nomogram predictions, we drew calibration plots and ROC curves at 1-, 3-, 5- years in the training set and at 1-, 3-, 5-, 10- in the testing set (Figure 5I-J).

### Association of clinical features, TMB, and CSC index with TM scores

**Figure 6:**
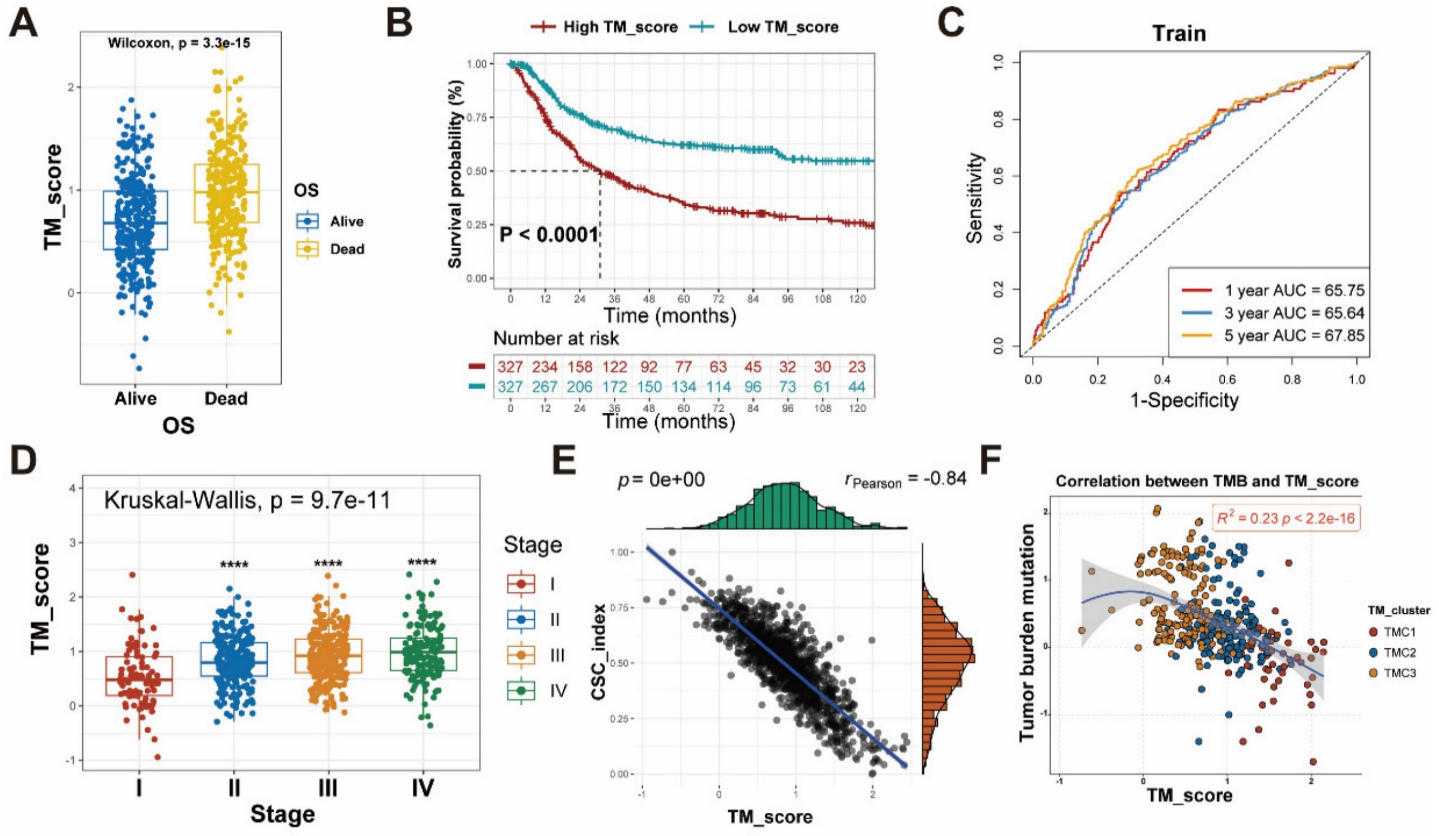
** Differences between high and low TM score groups. (A)** Patients with dead status achieved higher TM score. **(B-C)** Survival analysis with effective AUC revealed high TM score group had poorer prognosis. **(D)** The relationship between TM scores and clinical stages. **(E)** The relationship between TM scores and CSC index. **(F)** The relationship between TM scores and TMB.

**Figure 7:**
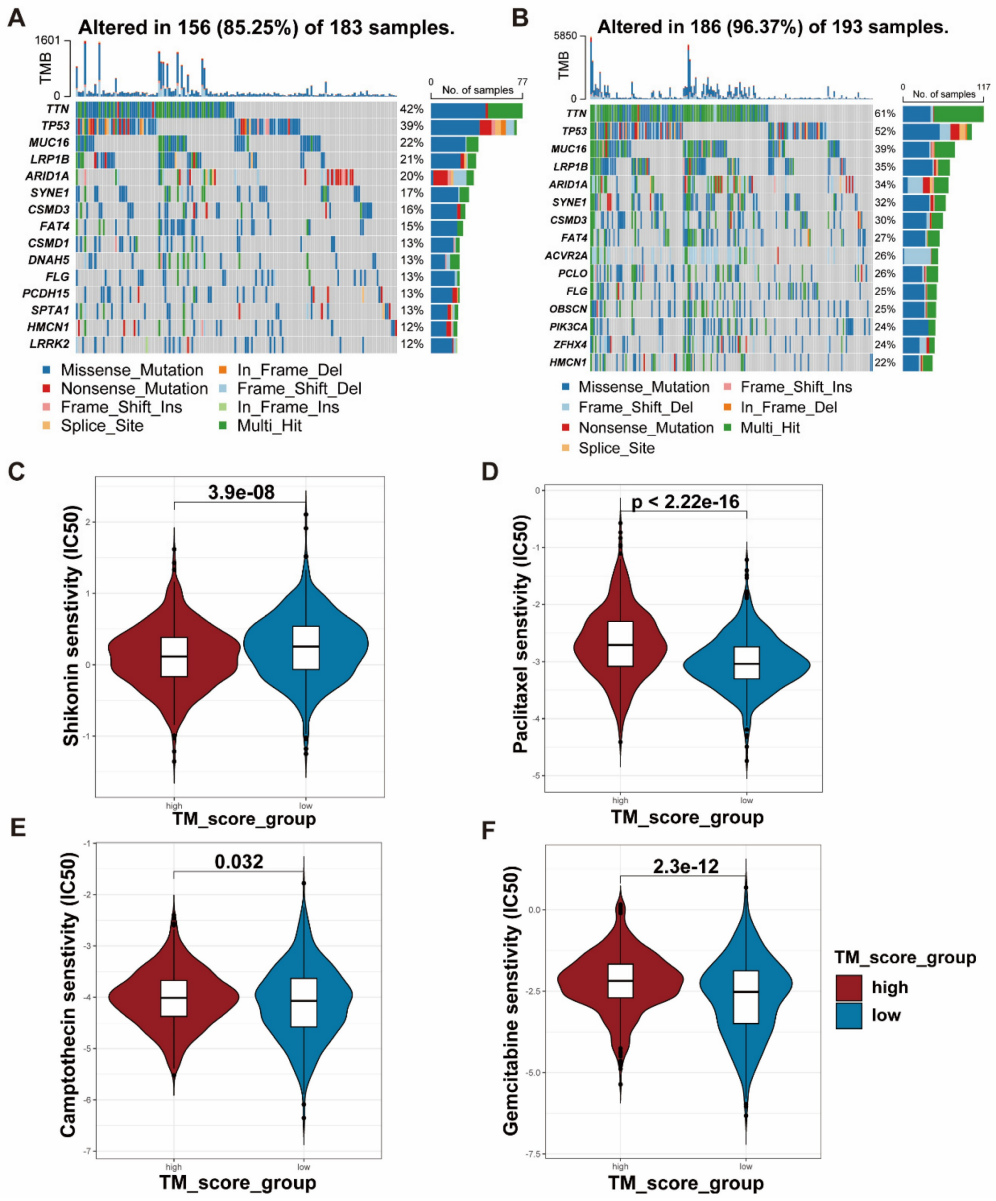
** TMB and drug sensitivity between high and low TM score groups. (A-B)** The relationship between TM scores and TMB shown with waterfall plots. **(C-F)** IC50 analyses revealed sensitivity of various drugs (Shikonin, Paclitaxel, Camptothecin and Gemcitabine) to the two TM score groups**.**

To examine the differences in TM scores between patients with various outcomes and clinical stages, we carried out a series of analyses. As a result, patients with high TM scores had lower survival probability and advanced clinical stage, whose efficiencies have been validated in the training and testing set (Figure 6A-D, Figure S5D-F). The CSC index was employed to describe the correlations of TM scores with cell stemness (Figure 6E). In terms of tumor mutation burden, the level of TM scores was negatively related to the TMB (Figure 6F), which prompted the possibility that the lower TM score might be sensitive to immunotherapy. The waterfall plots displayed the first 15 mutant genes of high and low TM score groups, respectively (Figure 7A, B). Moreover, drug sensitivity analysis showed that patients with high TM scores were more sensitive to paclitaxel, camptothecin, and gemcitabine, whereas shikonin may achieve better efficacy in patients with low TM scores (Figure 7C-F). The above results assisted in formulating personalized drug treatment plans for different patients.

### Validation of tumor-promoting effects of TUBB6 in gastric cancer

**Figure 8:**
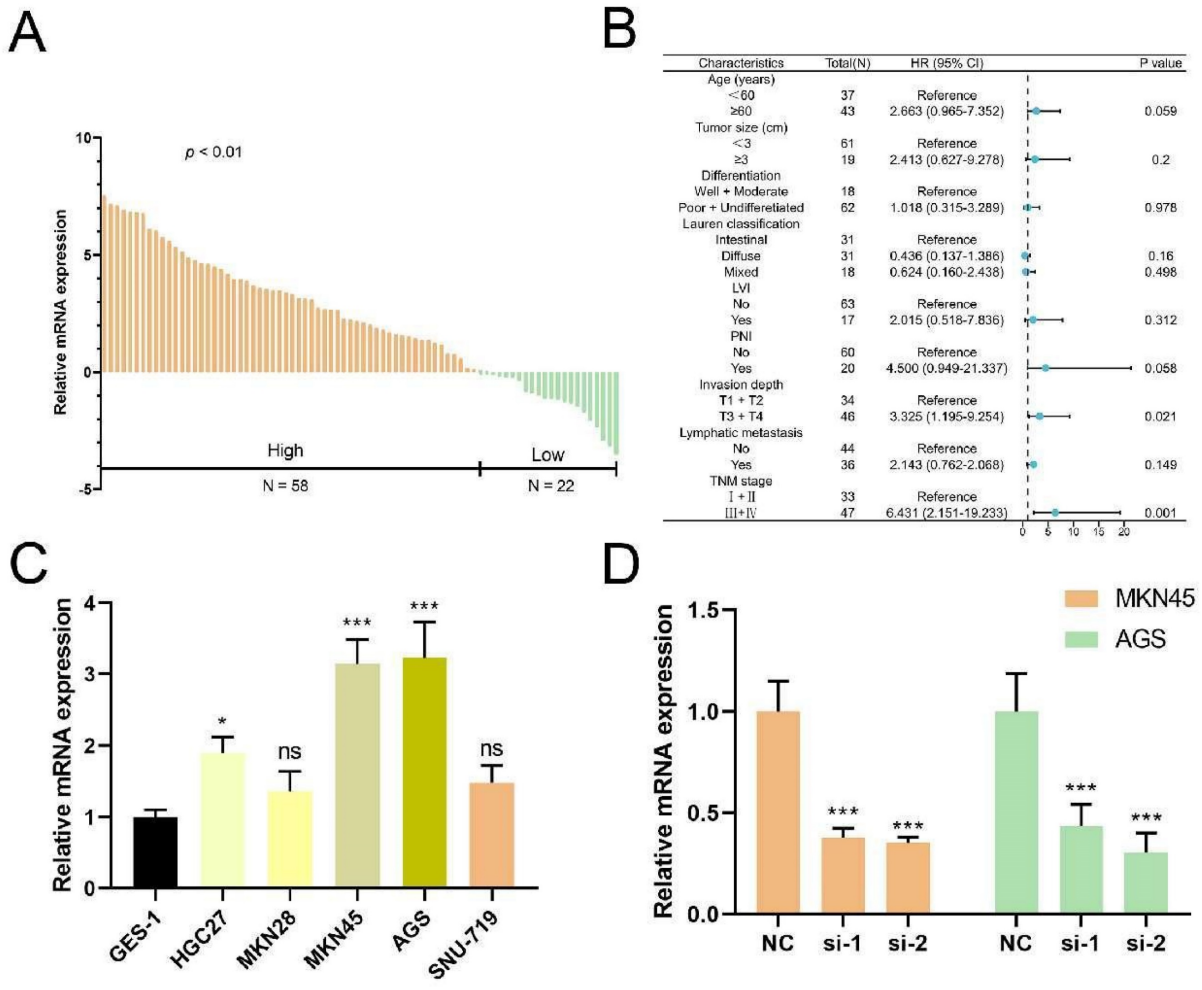
** TUBB6 is upregulated in GC and correlates with clinical features. (A)** qRT-PCR was used to detect the expression of TUBB6 in 80 GC tissues and paired adjacent normal tissues. **(B)** The cox regression analysis showed that high expression of TUBB6 was correlated with deeper invasion and advanced TNM stage. **(C)** qRT-PCR analysis was used to detect the TUBB6 expression in GC cells and normal gastric epithelium cell line (GES-1). **(D)** The knockdown efficiencies of siRNAs were validated by qRT-PCR.

**Figure 9:**
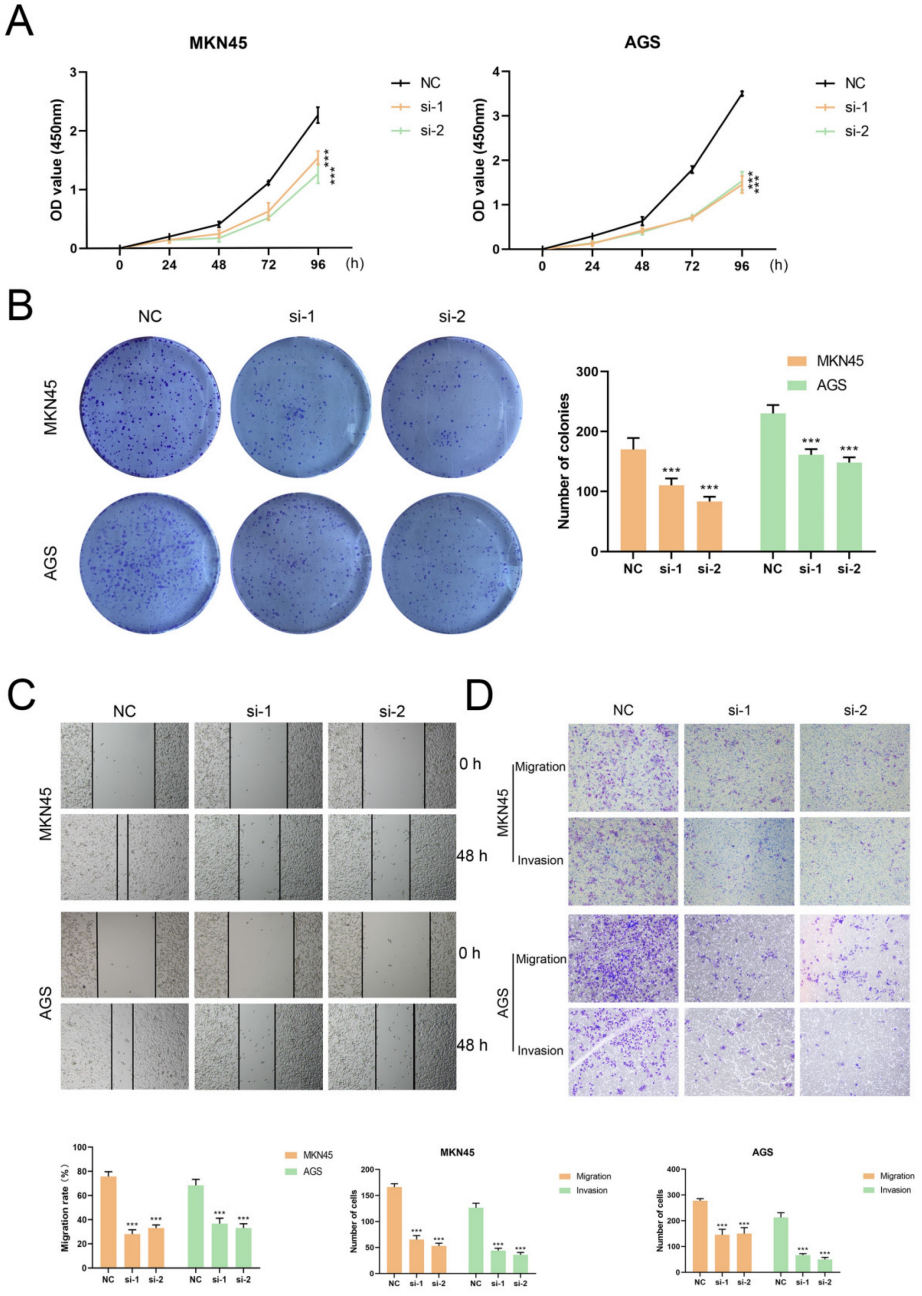
** TUBB6 could modulate the malignant behaviors of GC cells *in vitro*. (A)** The growth curves of cells were evaluated by CCK-8 assays after knocking down TUBB6 in MKN45 (right) and AGS (left) cells. **(B)** We conducted colony formation assays and statistical analysis to evaluate cell proliferation. **(C-D)** We conducted wound healing assays (G) and transwell assays (H) to evaluate cell migration and invasion.

Anchored in the aforementioned prognostic model (Figure 5, Table S7), 3 genes with positive coefficients were chosen. According to the expression level estimated by qRT-PCR (n = 32) (Figure S6), the gene TUBB6 was selected as our target gene which exhibited the highest relative expression in tumor tissues and demonstrated an obvious correlation with the bad survival prognosis. Foremost, we conducted quantitative real-time PCR (qRT-RCR) to validate the expression level of CYP19A1 in GC tissues and TUBB6 was identified as overexpressed in GC tissues compared with adjacent normal tissues (n = 80) (Figure 8A). Subsequent to the exploration of the relationships between the TUBB6 expression level and clinicopathological characteristics, it was shown by statistical analysis that the TUBB6 expression level was positively correlated with invasion depth and TNM stage (Figure 8B). To validate the effects of TUBB6, we performed qRT-PCR in several common GC cell lines and the normal gastric epithelium cell line (GES-1). The results showed that the expression of TUBB6 was markedly higher in some of the GC cell lines than that in GES-1, especially MKN45 and AGS (Figure 8C). Then, we selected the two aforementioned cell lines to carry out the following experiments and applied siRNAs to knock down TUBB6. The efficiencies of the siRNAs were validated by qRT-PCR (Figure 8D). Then, we made use of the two cell lines to perform CCK-8 assay, colony formation assay, wound healing assay, and transwell assay to explore the effects of TUBB6 having in cell proliferation, migration, and invasion. The results of the experiments were statistically analyzed and we obtained positive results which indicated that TUBB6 could promote the development and progression of GC (Figure 9A-D).

## Discussion

As is known to us, sustained cell division contributes telomeres to reaching an extremely short length that could trigger cell senescence and death, which foreshadows that the regulation of telomeres may offer unimaginable potential in the therapeutic treatments of multiple diseases 24. Leaving out telomere biology disorders in the traditional sense such as bone marrow failure syndrome and aplastic anemia 25, 26, malignant tumors have been thought closely related to telomere maintenance 5. Anchored in different ways to maintain telomere length, two possible approaches to fight against malignant tumors hold potential. TERT, the core protein subunit of the telomerase, was one of the potential therapeutic targets. Altering the expression of TRET via transcriptional regulation and alternative splicing could make sense for beating cancer 27. For ALT tumors, the inhibition of some specific molecules, such as PGC1β, SOD2, and ATR may be another pathway 5, 28, 29. As we can see, some drugs targeting telomerase have been in clinical trials, including Imetelstat and GV1001 30, 31. Additionally, previous studies have published that TERRA could function as a marker for screening ALT cancers and the correlations between cell cycle proteins and the length of telomeres 32, 33. Apart from telomere maintenance, human telomerase reverse transcriptase was known to play roles in EMT, migration and invasion, angiogenesis, and activation of fibroblasts in malignant tumors 34-36, which may promote the formation of the immunosuppressive environment homeostasis of the TME. Mechanically, telomerase reverse transcriptase plays the role of a co-activator in the process of activating multiple signaling pathways, such as Wnt/β-catenin, PI3K/Akt, and cGAS-STING pathway 37-39. Considering that telomerase reverse transcriptase might be regulated by some signaling pathways, a complex network of feedback mechanisms seems to exist between telomere maintenance and TME 40, which emphasizes the importance of telomere maintenance in cancers.

In our study, three clusters of GC patients were identified by the use of unsupervised clustering anchored in the expression of genes correlated with telomere maintenance. Significant differences were shown in clinical characteristics, biological pathways, immune infiltration, and genomic status among the three clusters. In terms of biological pathways, TMC1 correlated with EMT, WNT, and TGF-beta pathway, while TMC3 was linked to cell cycle, MYC, and DNA repair. As classical pathways activated in cancers, WNT and TGF-beta pathways were published to affect telomere maintenance as well 41, 42. MYC has been reported to interact with F-actin as well as TRF1 and take part in telomere maintenance and DNA repair 43, 44. From the perspective of TME, though various TMCs had different respective immune infiltration cells, TMC1 got relatively higher stromal scores and had more Treg and M2 macrophage cells. M2 macrophages, known as tumor-associated macrophages, could alter the state of transcriptional activation of functionally critical cytokines and regulate metabolic pathways 45. Abundant Treg cells could infiltrate into tumor tissues and predicted poor prognosis 46, which was in line with the effects of TMC1 on clinical outcomes. With respect to TMB, despite the similar mutational spectra they share, TMC3 had significantly more TMB than TMC1. This point has been repeatedly confirmed by the analysis of drug susceptibility, which showed that TMC3 could benefit more from the majority of immune or chemotherapy agents.

TUBB6 gene is located at chromosome 18p11.21 and codes the protein positioned in the microtubule that is known to be involved in microtubule cytoskeleton organization and mitotic cell cycle. In addition, TUBB6 was predicted to promote GTP binding activity. Many years of research revealed the intricate relationship between TUBB6 and malignant tumors. Cytoskeletal genes, including TUBB6, were found related to HBx-induced hepatocarcinogenesis as early as 2007 47. Colorectal cancer-related research indicated the correlations between poor outcomes, microsatellite instability, and TUBB6 48, 49. Moreover, TUBB6 was published to promote the hypermethylation of CpG islands in the promoter region of tumor-related genes in GC, hepatocellular carcinoma, and high-grade serous ovarian carcinoma 50-52. It has also been shown by large numbers of bioinformatic analyses that TUBB6 was associated with cell migration, pyroptosis, and muscle-invasion in non-small cell lung cancer, clear cell renal cell carcinoma, and bladder cancer, respectively 21, 53, 54. When it comes to clinical treatments, TUBB6 was proven to affect prognosis and drug-resistance in various tumors 55, 56. The aforementioned study implied the role TUBB6 plays in cell malignant behaviors, which opens the possibility of attaching TUBB6 to telomere maintenance. Concurrently, there exists no study validating the function of TUBB6 in GC.

Despite unique merits, limitations in our study ought not to be neglected. First of all, telomere maintenance could be regulated in various manners and we have not differentiated them in the analytical process. Secondly, we preliminarily validated the expression level and biological function of TUBB6 in GC. However, the specific target molecules and mechanisms remained unexplored. Then, data applied in our study was obtained from public databases and our own sequencing data may result in a more persuasive conclusion. Finally, it is a pity that we have not found specific methods to translate our research outcomes to the clinic apart from the TM score.

To sum up, different telomere maintenance-related molecular subtypes of GC were identified, followed by evaluation of clinical characteristics and tumor microenvironment of each subtype. Additionally, the TM score was established to predict the prognosis of GC patients. TUBB6 was preliminarily validated to be upregulated in GC and promote malignant behaviors. Our consequences provided effective guidelines for future diagnosis and treatment of GC.

## Supplementary Material

Supplementary figures and tables.

## Abbreviations

TMCs: Telomere maintenance-associated clusters
TME: Tumor microenvironment
TMB: Tumor mutation burden
TM: Telomere maintenance
TMGs: Telomere maintenance-related genes
