# Supplementary material for: Cross-talk of Three Molecular Subtypes of Telomere Maintenance Defines Clinical Characteristics and Tumor Microenvironment in Gastric Cancer
Source: J Cancer. 2024 Apr 15;15(10):3227–41. doi: 10.7150/jca.92207 (PMC11064253; doi:10.7150/jca.92207)
Supplement: Supplementary file 1 — Supplementary figures and tables. [file jcav15p3227s1.zip › Supplementary Figures.docx]

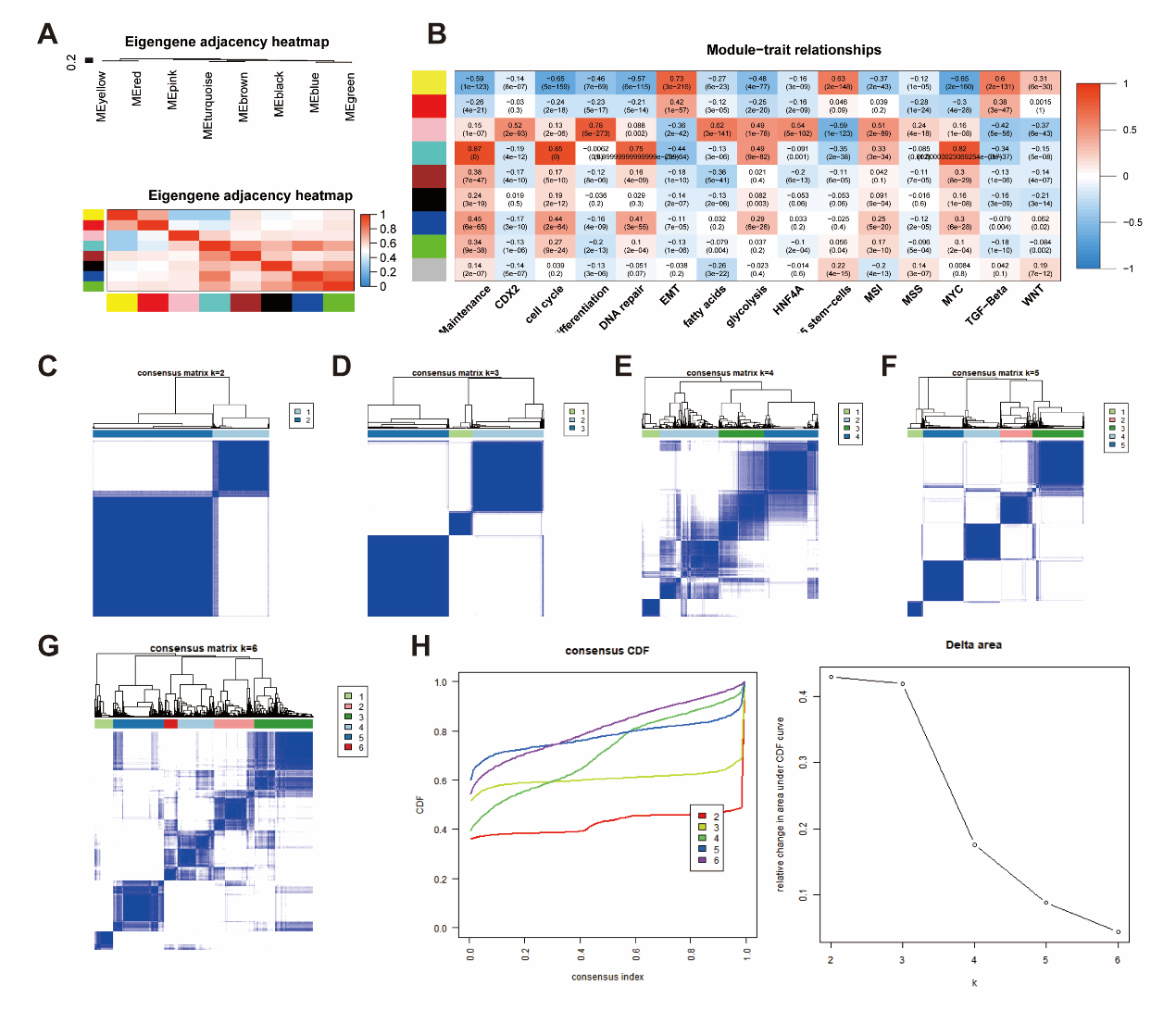


**Supplementary Figure S1: WGCNA screening suitable TM-related genes for construction of TM clusters.** **(A)** The association between different gene modules. **(B)** The relationship between biological pathways and gene modules. **(C-G)** The heat map of consensus clusters (k= 2-5). **(H)** The CDF and delta index of consensus clustering.


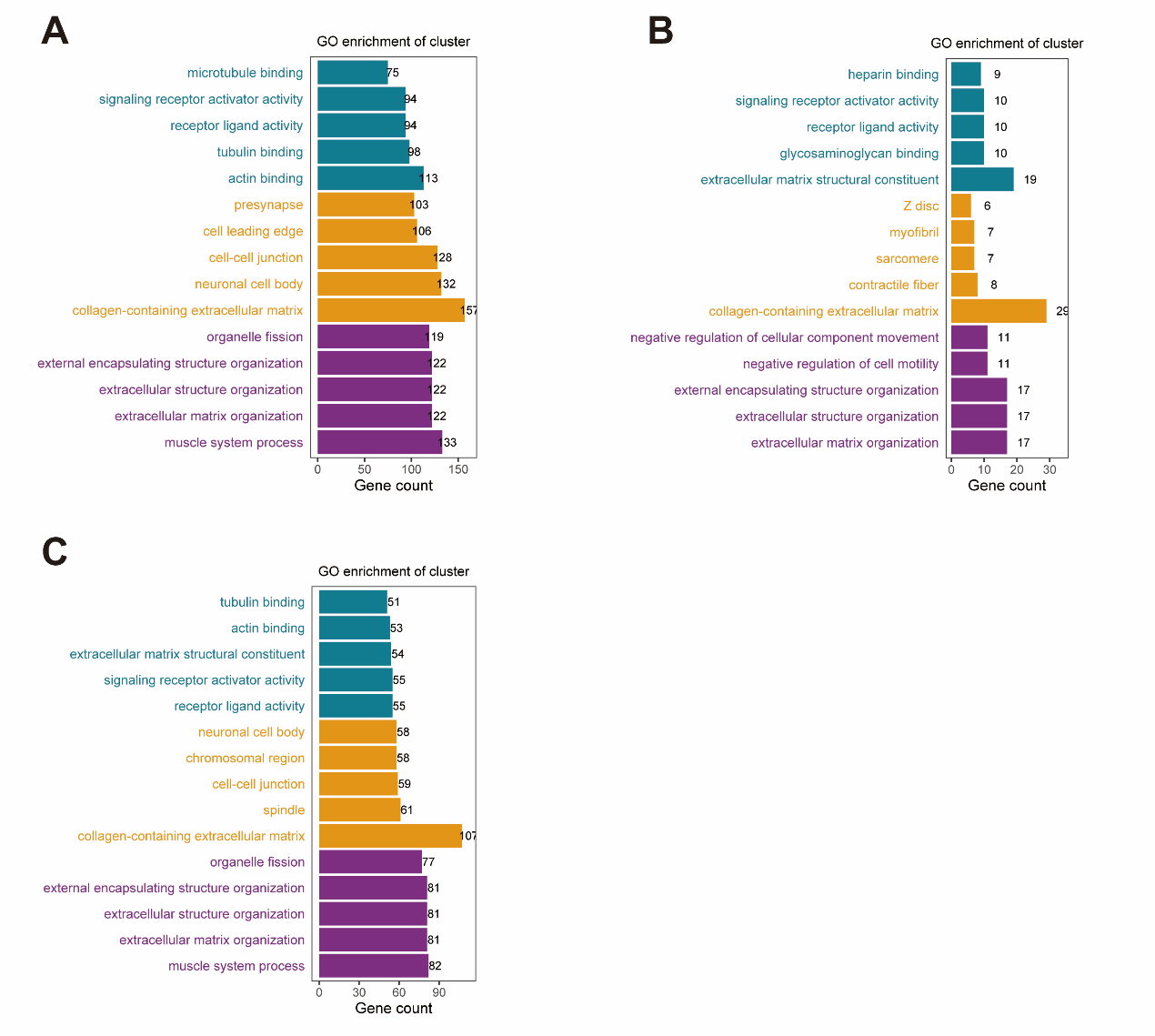


**Supplementary Figure S2: GO analysis of the three clusters.** **(A)** GO analysis of the three TM clusters, respectively.


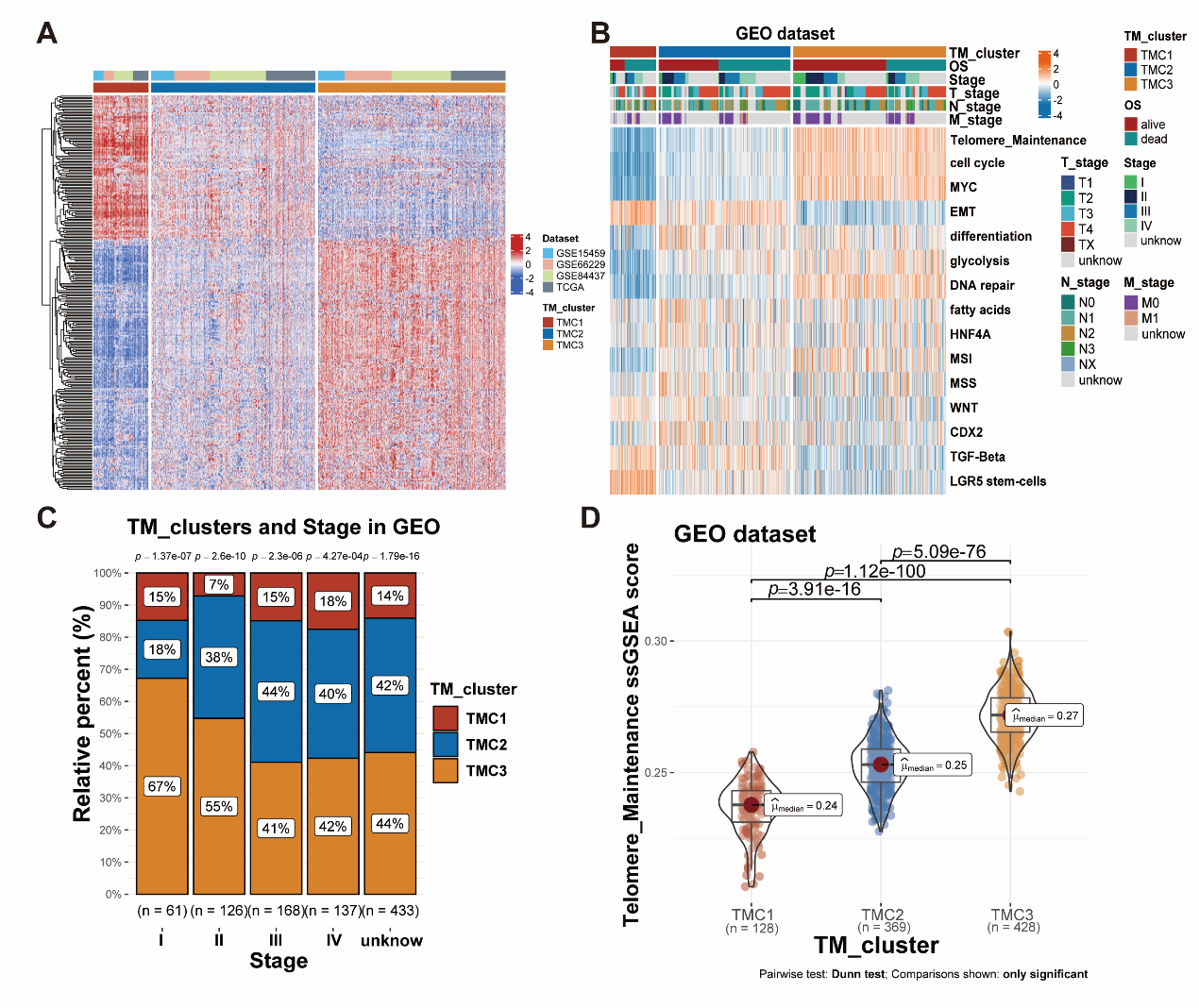


**Supplementary Figure S3: Clinical traits of TM clusters in meta-GEO datasets.** **(A)** The expression of 282 TM-related genes in TM clusters. **(B)** The distinctions between TMCs were discovered by examining clinical characteristics and 15 pathways assessed by ssGSEA in meta-GEO datasets. **(C)** Proportion of TMCs in different clinical stages in meta-GEO datasets. **(D)** TMCs’ respective TM ssGSEA scores uncovered that TMC3 scored the highest while TMC1 the lowest in meta-GEO datasets.


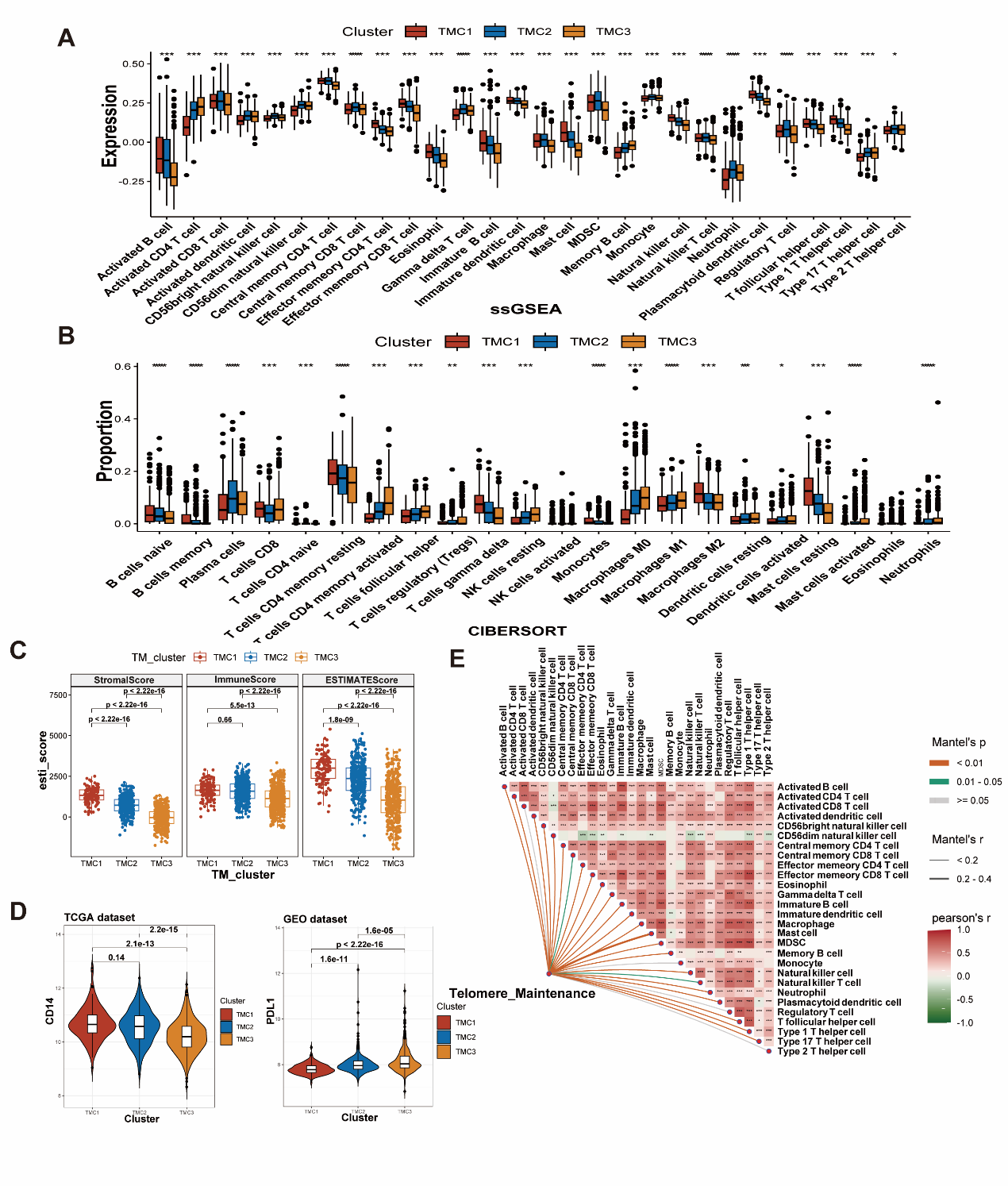


**Supplementary Figure S4: Exploration of TME in TMCs in meta-GEO datasets.** **(A)** ssGSEA analysis revealed the different infiltration status of immune cells in TMCs. **(B)** Cibersort analysis exhibited the Immune cells’ proportion in TMCs. **(C)** ESTIMATE algorithm assessed immune and stromal scores in TMCs. **(D)** TM was calculated using association analysis on different immune cells. **(E)** Differential expression of PDL1 and CD14 in TMCs (* p <0.05, ** p <0.01, *** p <0.001).


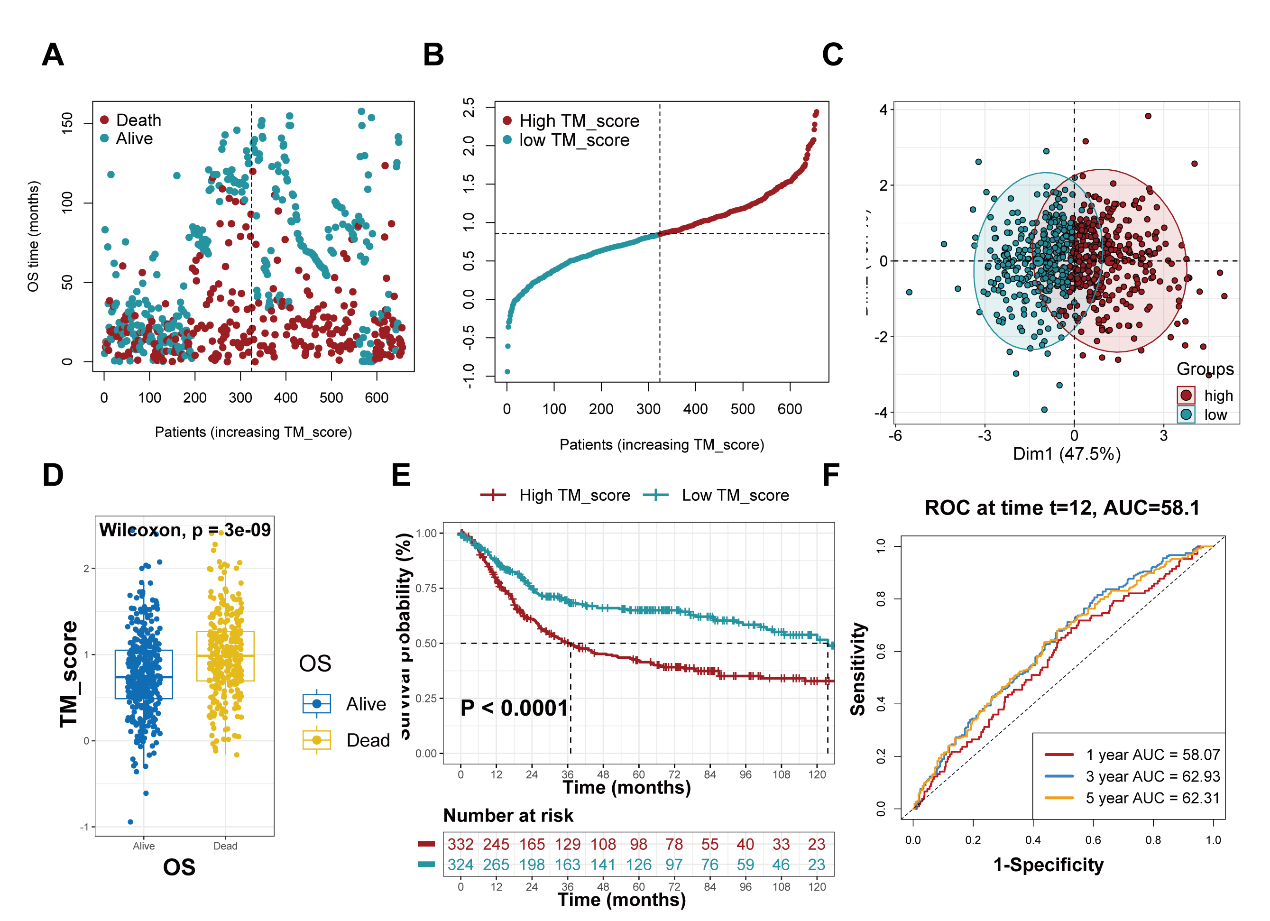


**Supplementary Figure S5: Validation of TM score in test set.** **(A-B)** Distribution of TM scores in patients with different OS status in test set. **(C)** PCA analysis exhibited the distribution between the high and low TM score groups in test set. **(D)** Patients in test set with dead status achieved higher TM score. **(D)** TM was calculated using association analysis on different immune cells. **(E-F)** Survival analysis with effective AUC revealed high TM score group had poorer prognosis in test set.


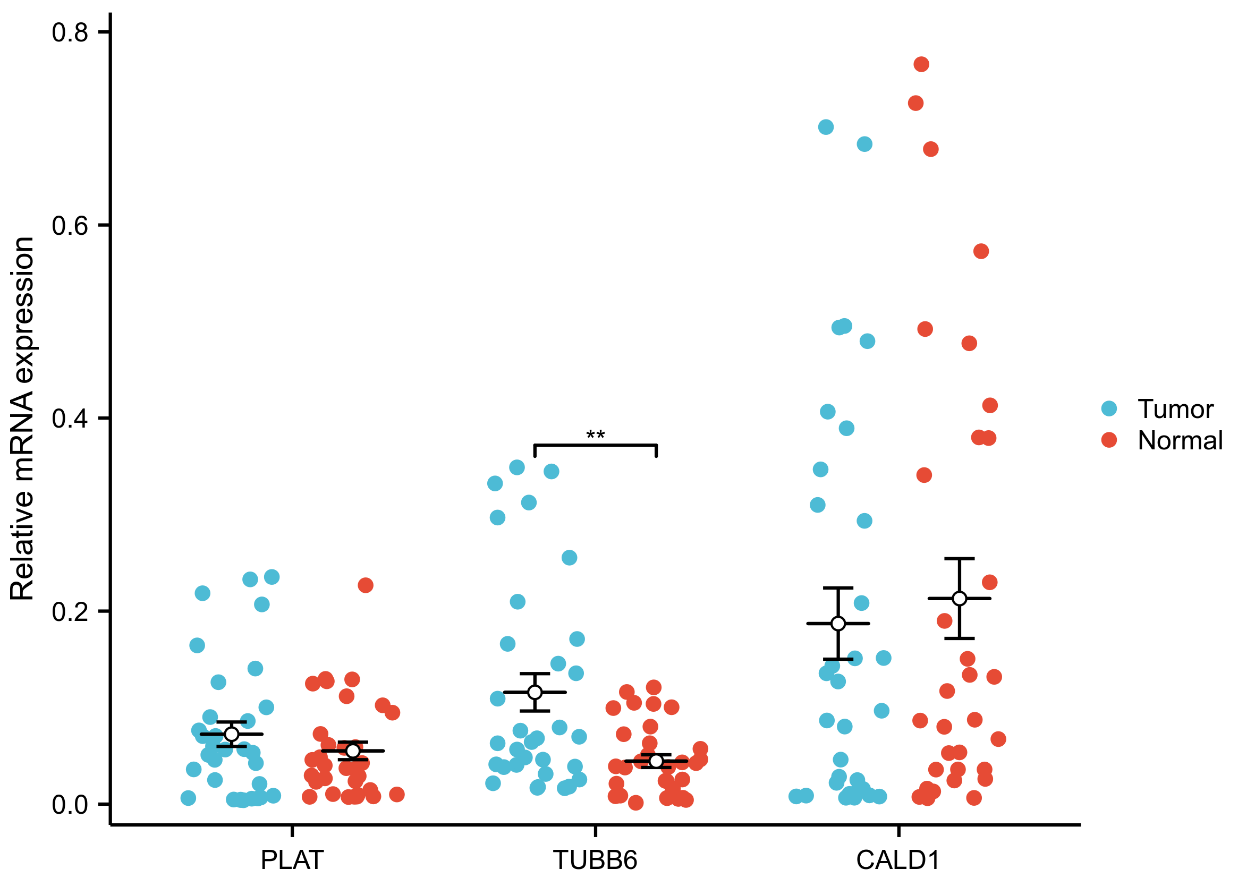


**Supplementary Figure S6: qRT-PCR analysis of PLAT, TUBB6, and CALD1 (* p <0.05, ** p <0.01, *** p <0.001).**
